# Punishing second-order free riders before first-order free riders: The effect of pool punishment priority on cooperation

**DOI:** 10.1038/s41598-017-13918-8

**Published:** 2017-10-30

**Authors:** Hiroki Ozono, Yoshio Kamijo, Kazumi Shimizu

**Affiliations:** 1 Faculty of Law, Economics and Humanities, Kagoshima University, 1-21-24, Korimoto, Kagoshima-shi, Kagoshima-ken, 890-0065 Japan; 2 School of Economics and Management, Kochi University of Technology, 2-22, Eikokuji-Cho, Kochi-Shi, Kochi-Ken, 780-8515 Japan; 3 School of Political Science and Economics, Waseda University, 1-6-1, Nishi-Waseda, Shinjuku-ku, Tokyo, 169-8050 Japan

## Abstract

Second-order free riders, who do not owe punishment cost to first-order free riders in public goods games, lead to low cooperation. Previous studies suggest that for stable cooperation, it is critical to have a pool punishment system with second-order punishment, which gathers resources from group members and punishes second-order free riders as well as first-order free riders. In this study, we focus on the priority of punishment. We hypothesize that the pool punishment system that prioritizes second-order punishment is more likely to achieve cooperation than the system that prioritizes first-order punishment, because the former is more likely to obtain sufficient punishment resources. In the experiments, we compare four pool punishment systems: 1To2 (first-order punishment to second-order punishment), 2To1 (second-order punishment to first-order punishment), 1ONLY (first-order punishment only), and 2ONLY (second-order punishment only). We find that the 2To1 and 2ONLY systems can receive more support than the 1To2 and 1ONLY systems and only the 2To1 system can achieve high cooperation. However, the effect of priority of second-order punishment is observed only when the punishment ratio (PR) is low (Experiment 1), not high (Experiment 2), in which the punishment resource is relatively abundant.

## Introduction

Solutions to public goods problems, such as payment for public TV programs and preservation of the natural environment, are one of the most important issues for human society^1,2^. The difficulty of providing such public goods is well formulated by using public goods games (PGGs). In a PGG, individuals in a group decide to contribute to the common pool, and the total amount of the contribution is shared equally by the group members. In this situation, free riders, who do not contribute but receive benefits from others’ contributions, are more beneficial than cooperators are, and thus, a group faces a serious difficulty in providing public goods: everyone is worse off than they would have been had they all contributed fully to the public goods. Many studies have suggested that peer punishment could solve this free-rider problem because the benefits of free riders could be lower than the benefits of cooperators if free riders are punished seriously^3–7^. However, if peer punishment is costly because of, for example, possible retaliation^8,9^ and energy expenditure, then cooperators who do not punish will be better off than cooperators who punish will be. Thus, the provision of punishment of noncooperators is itself a collective act that can suffer another free-rider problem, which is sometimes called the second-order free-rider problem^8,10^. As a result, second-order free riders, who do not owe the cost of punishment, can lead their group to a low cooperation level because a sufficiently high cost is not imposed on first-order free riders (noncooperators) to encourage them to refrain from free riding. The first-order punishment can be maintained by the punishment of the second-order free riders (nonpunishers), but this second-order punishment is costly and itself amounts to another collective act^11–13^. Speaking in more general terms, to solve the first-order free-rider problem, we should resolve the second-order free-rider problem. However, to solve the second-order free-rider problem, we should resolve the third-order free-rider problem, and thus, the situation falls into infinite retrogression. Peer punishment appears vulnerable to infinite regression. In addition to this theoretical problem, a recent anthropological survey revealed that peer punishment to free riders is very rare in small-scale societies, which are similar to an evolved environment^14^.

By considering the vulnerability of peer punishment, some researchers have focused on the pool punishment system, in which individuals opt to support a punishment system (e.g., a police force) and the system punishes free riders by using resources (“punishment fund”) supported by members^15–17^. Sigmund *et al*.^15^ compared peer punishment with pool punishment and mathematically showed that the pool punishment system is more stable than the peer punishment system only when the system punishes not just the first-order free riders (noncooperators) but also the second-order free riders (nonsupporters), who do not contribute to the punishment system. Traulsen *et al*.^17^ examined the pool punishment system in a laboratory experiment, which showed that participants tend to select pool punishment over peer punishment. In addition, the authors reported that systems with second-order punishment increased the number of people supporting the system, and thus, high cooperation is more likely to be achieved compared to the condition with only first-order punishment because the system with second-order punishment has sufficient resources to punish noncooperators.

In this study, we focus on the priority of first- and second-order punishment. Since punishment itself is costly and punishment resources are finite, it is important to prioritize punishment, namely, which kind of free riders (“noncooperators” or “nonsupporters”) should be punished first, for the system to use the limited punishment resources effectively. However, previous studies about the pool punishment system with second-order punishment have ignored this question, probably for simplicity. For example, in the experiment of Traulsen *et al*.^17^, in which group size is five, each of first- and second-order free riders are punished by 1 token when at least one member of the group decides to provide 0.5 tokens to the pool punishment system. In this situation, if there is only one supporter in the group, the pool punishment system can punish all four other noncooperators or nonsupporters by only 0.5 tokens each, but it seems especially effective to punish all of them with such a limited resource compared to the settings of previous studies (see the metaanalysis of Balliet^18^).

Considering the priority of punishment, one may intuitively think that first-order free riders (noncooperators) should be punished first because the aim of pool punishment system is establishment of cooperation. However, we show that that intuition fails. We predict a group can establish a high cooperation level more easily in the system that prioritizes second-order punishment than in the system that prioritizes first-order punishment, because the former can construct a “punishment fund” more easily and lead to cooperative behavior.

**Figure 1:**
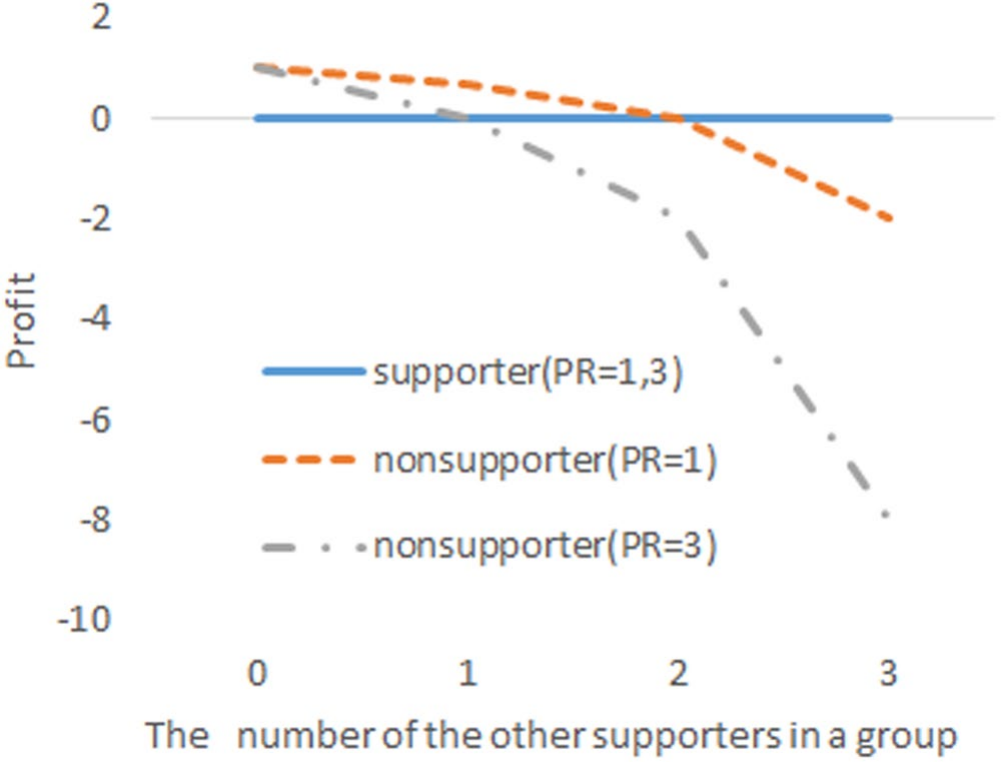
Profit of supporters and nonsupporters with PR = 1 and PR = 3 in the pool system with only second-order punishment.

By comparing a system with only first-order punishment and that with only second-order punishment, we can clarify the reason for this prediction in detail. On the one hand, in the system with only first-order punishment, a group suffers from the second-order free-rider problem because the second-order free riders (nonsupporters) are not punished. This leads to a shortage of resources to punish the first-order free riders (noncooperators), and thus, high cooperation is not achieved. In other words, the system with only first-order punishment is a social dilemma game and its rational outcome is only noncooperation and nonsupport by all members. On the other hand, in the system with only second-order punishment, the payoff structure does not always suffer from the free-rider problem, because it might be more beneficial for individuals to support the system in certain situations. We introduce a simple system with second-order punishment played by four members. First, each of the four members has 1 token and decides whether to support the system, that is, to provide this token for the system. The system punishes nonsupporters by using the resources pooled by the supporters. Supporters are never punished, and thus, their profit is zero. The profit of nonsupporters depends on the number of supporters and the punishment ratio (PR), which describes the sum of the profit reductions for the targeted members relative to the amount of support for the pool punishment system. Figure 1 shows the profit of supporters and nonsupporters with PR = 1 and PR = 3.

It is more beneficial for members to support the system than not to support it when two or three other members in PR = 3 and three in PR = 1 support the system. When no members in PR = 3 and no or one other member in PR = 1 supports the system, it is more beneficial for them not to support the system. This payoff structure suggests that the system with only second-order punishment is neither a PGG nor a social dilemma game, but a coordination game^19–21^, in which the same behavior as the other members is more beneficial, and both support by all members and support by no members are Nash equilibria. Therefore, in the system with second-order punishment, it is possible that people contribute sufficiently to the punishment system. Moreover, previous studies about coordination games in laboratory experiments have suggested that participants tend to choose risk-averse options^20–23^. In the system with only second-order punishment, the contribution for the punishment system is risk-averse choice, because supporters have no risk of being punished by the system. For this reason, the equilibrium of all supporters will be more likely to occur than the equilibrium of all nonsupporters.

Based on this argument, we consider the system equipped with both first- and second-order punishment. In the system that prioritizes second-order punishment, we predict that members tend to support the system more and the system can have sufficient resources to punish noncooperators in the PGG, which results in achievement of high cooperation. By contrast, in the system that prioritizes first-order punishment, the members will be less likely to support the system, which cannot punish noncooperators sufficiently and results in low cooperation.

In our study, we compare four pool punishment systems, which have only first-order punishment (1ONLY), only second-order punishment (2ONLY), first-order to second order punishment (1To2), and second-order to first-order punishment (2To1). We investigate which system enjoys sufficient support and establishes high cooperation in the PGG.

The experiment consists of a PGG stage and a pool punishment stage. As one set, these two stages are repeated 15 times among four group members with partner treatment. In the PGG stage, each member is given 20 tokens and decides how many tokens to contribute to his/her group from 0 to 20. The total contributions are multiplied by 1.6 and distributed equally to all members. In the pool punishment stage, each member is given another 9 tokens and decides how many tokens to provide (support) for the pool punishment system from 0 to 9. After this decision, the system punishes the members according to the rule of each condition. In the 1ONLY condition, the least cooperator in the group is punished first and subsequently, the second least is punished; this process continues until the punishment resources are exhausted. In the 2ONLY condition, the least supporter in the group is punished first and subsequently, the second least is punished; this process continues until the punishment resources are exhausted. The 1To2 and 2To1 conditions are combinations of the 1ONLY and 2ONLY conditions. In the 1To2 condition, if the punishment resources remain after the punishment for noncooperators, the remaining punishment resources are used for the punishment of nonsupporters. In the 2To1 condition, the order of punishment described above is reversed; nonsupporters are punished first and noncooperators second. Full cooperators, who contributed 20 to the group, are never punished in the first-order punishment and full supporters, who provided 9 to the system, are never punished in the second-order punishment. The punishment system of the four conditions is similar to the relative punishment (penalty) system employed by other experimental studies^12,24^.

Throughout all conditions, our pool punishment system uses resources provided by the members to reduce the tokens of the free riders, that is, less cooperators and/or less supporters. The system reduces the target’s tokens to zero and the system spends its resources, which equals the amount of tokens the system reduced from the target. For example, the system with 1ONLY has 30 tokens as punishment resources provided by the member before executing punishment. When the least cooperator, who owned 24 tokens, is punished, the 24 tokens are reduced from the least cooperator and the system spends 24 tokens as well. As a result, 6 tokens (=30–24) can be used to the second least cooperator. If the punishment resources are exhausted, the system can no longer punish. This system needs more resources for the larger number of free riders. We consider that this punishment system meets the requirements in real life: as the punishment resources are limited, the system always faces a risk of shortage to punish free riders sufficiently.

We manipulate the PR to examine the effects of the amount of punishment resources on people’s cooperation. In Experiment 1, the PR is 1, which means that the total amount provided by all supporters becomes identically the punishment resources. In Experiment 2, the PR is 3, which means that the total amount provided by all supporters is multiplied by 3 and that becomes the punishment resources. The priority of punishment is critical when the punishment resource is scarce. Under scarcity, only the system with 2To1 can obtain sufficient support because the system punishes nonsupporters first, and thus, the system can have sufficient resources to punish noncooperators in the PGG, which results in the achievement of high cooperation. The system with 1To2 suffers from a shortage of punishment resources to punish nonsupporters because the system punishes nonsupporters only after the system uses the limited resources to punish noncooperators. The shortage of resources must be more likely to occur under a low PR. If the PR were higher, the system might be able to punish nonsupporters even after punishing all noncooperators because the punishment resources constraint is mitigated by the high PR. Therefore, the priority of first- and second-order punishment is not as important when the PR is high. In this study, we examine this prediction by comparing a low PR, 1, and a high PR, 3.

Another important analysis concerns the “surplus of the pool punishment system,” which is defined as the final residue that has not been used to punish. Historically, a punishment system was governed by a specific governor or a few governors of the group, such as headmen in villages, lords of manors, or kings of nations. They could obtain the surplus, and thus, might choose more profitable punishment system for themselves^25^. Therefore, analysis of the surplus has implications for institutional choice by governors.

We propose the following two hypotheses.

H1: In 2ONLY and 2To1 conditions, in which second-order punishment has a priority, the participants are more likely to support the system than in 1ONLY and 1To2 conditions, in which first-order punishment has a priority.

H2: High cooperation is more likely to be achieved in the 2To1 condition than in the other three conditions.

H3: Difference of cooperation level between 2To1 condition and 1To2 is observed more clearly under a low PR than under a high PR.

## Results

### Experiment 1

In Experiment 1, PR was 1. The data were analyzed at the group level to take into account interdependence of outcomes for members of a given group. All the multiple comparison results of the nonparametric analyses were corrected by Bonferroni’s method.

**Figure 2:**
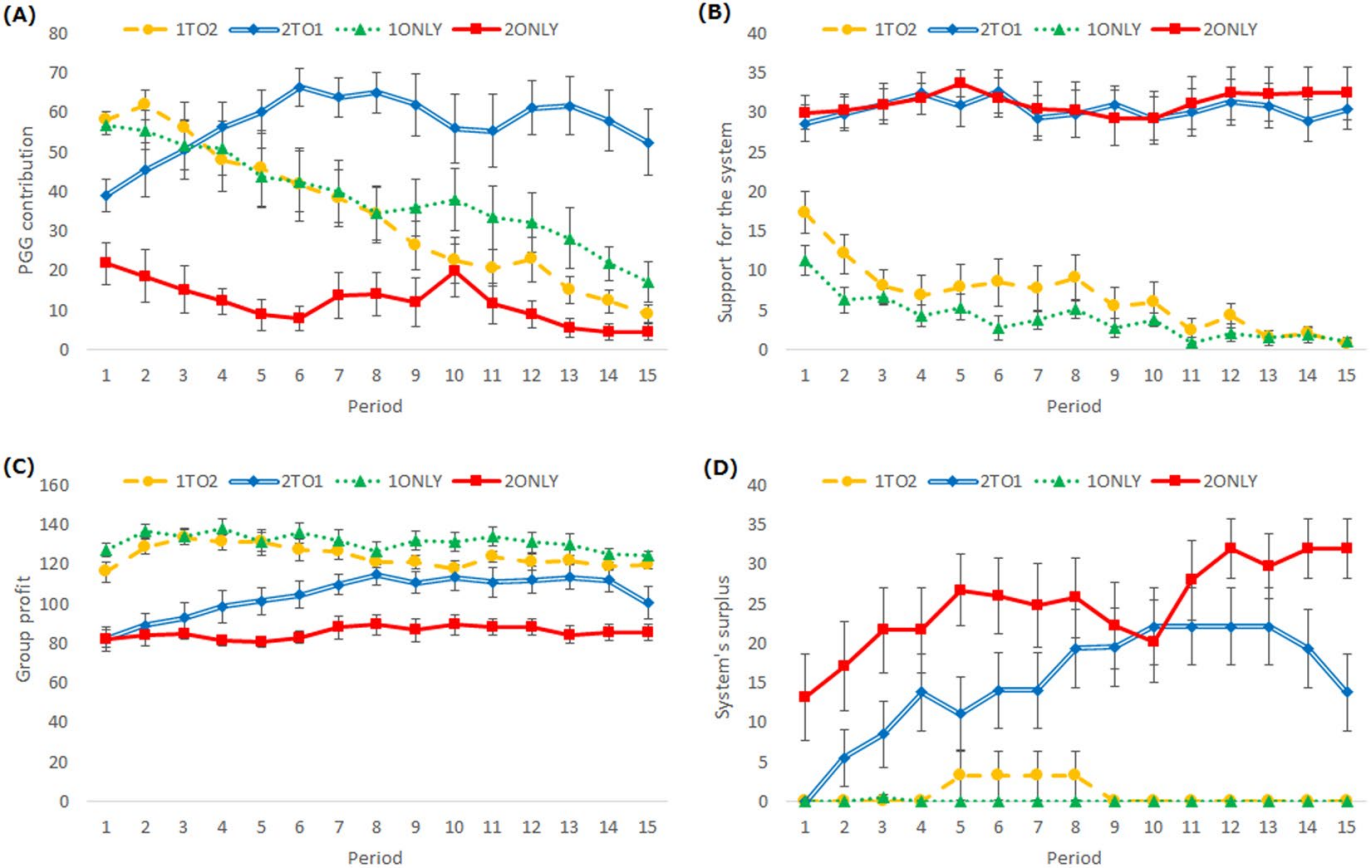
(**A**) Total PGG contribution, (**B**) total support for the system, (**C**) group profit, and (**D**) system’s surplus (=total support − total use for punishment) over 15 periods of play under four conditions in Experiment 1 (error bars denote standard errors).

The total PGG contribution, total support for the system, group profit, and system’s surplus (=total support – total use for punishment) were calculated for each period (Fig. 2). We compared the average of each index of the second half of the periods, that is, from the 8th to 14th periods, when participants had sufficient time to settle on their conditions. We excluded the final (15th) period from the data, because all participants knew it would be the last, and therefore, they were considered to behave differently. Mann–Whitney U-tests were conducted to determine whether a difference existed between the conditions.

Participants in the 2To1 and 2ONLY conditions supported the system more than did those in the 1ONLY and 1To2 conditions (2To1vs1ONLY, *U* = 0, *p* < 0.001; 2To1vs1To2, *U* = 3, *p* < 0.001. 2ONLYvs1ONLY, *U* = 2, *p* = 0.001; 2ONLYvs1To2, *U* = 3, *p* = 0.001). There were no significant differences in the other comparison of conditions (2To1vs2ONLY, *U* = 56.5, *p* = 1.00; 1To2vs1ONLY, *U* = 41.5, *p* = 1.00). These results are consistent with H1, that is, participants support the system more in the system that prioritized second-order punishment. Moreover, high cooperation was more likely in the 2To1 condition than in the 1To2 (*U* = 18.5, *p* = 0.007), 1ONLY (*U* = 21, *p* = 0.027), and 2ONLY (*U* = 5, *p* < 0.001) conditions. There were no differences in the other comparisons of conditions (1To2vs1ONLY, *U* = 39.5, *p* = 1.00; 1To2vs2ONLY, *U* = 27, *p* = 0.571; 1ONLYvs2ONLY, *U* = 14, *p* = 0.061). These results are consistent with H2, that is, high cooperation is more likely in the 2To1 condition than in the other three conditions.

Group profit was lower in the 2ONLY condition than in the 1To2 (*U* = 7, *p* = 0.003) and 1ONLY (*U* = 4, *p* = 0.002) conditions, while there were no differences in the other comparison of conditions (1To2vs1ONLY, *U* = 32, *p* = 0.687; 1To2vs2To1, *U* = 66, *p* = 1.00; 1ONLYvs2To1, *U* = 46, *p* = 1.00; 2ONLYvs2To1, *U* = 23, *p* = 0.091). Although the 2To1 condition could achieve high cooperation, the condition could not obtain higher profit than the other conditions, because punishment cost to maintain high cooperation was higher in the 2To1 condition than in the other conditions and support for the system continued even after full contributions were achieved. This low efficacy in the 2To1 condition is consistent with previous studies of the pool punishment system^15,17^. The system’s surplus was higher in the 2ONLY and 2To1 conditions than in the 1ONLY and 1To2 conditions (2To1vs1ONLY, *U* = 20, *p* = 0.010; 2To1vs1To2, *U* = 25, *p* = 0.011; 2ONLYvs1ONLY, *U* = 5, *p* = 0.001; 2ONLYvs1To2, *U* = 6, *p* < 0.001). There were no significant differences in the other comparison of conditions (2To1vs2ONLY, *U* = 52, *p* = 1.00; 1To2vs1ONLY, *U* = 50, *p* = 1.00). This suggests that the system prioritizing second-order punishment could receive more surplus, which might result in more profit for the system’s governor(s).

### Experiment 2

In Experiment 2, the PR moved from 1 to 3. This experiment investigated whether the effect of the priority of the first- and second-order punishment would be the same even with higher punishment efficiency. We predicted that the priority was not critical for cooperation when punishment resources were relatively sufficient (H3). We eliminated the 2ONLY condition in Experiment 2. The 2ONLY condition in Experiment 1 shows that the system was supported fully and could yield large profit, but the group could not achieve high cooperation. It is natural to assume that this result would be replicated in Experiment 2.

**Figure 3:**
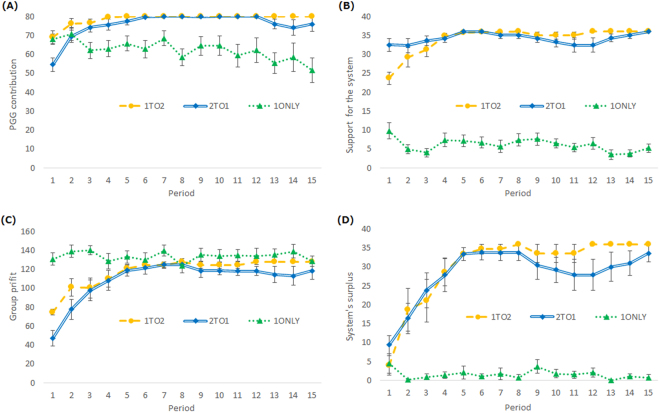
(**A**) Total PGG contribution, (**B**) total support for the system, (**C**) group profit, and (**D**) system’s surplus (= total support − total use for punishment) over 15 periods of play under three conditions in Experiment 2 (error bars denote standard errors). In the 1To2 condition, all the groups achieved full contribution to their group from the 4th to 15th periods as well as full support for their system in the 8th, 12th, 13th, 14th, and 15th periods. In the 2To1 condition, all the groups achieved full contribution to their group in the 7th, 8th, 10th, 11th, and 12th periods as well as full support for their system in the 5th, 6th, and 15th periods. Thus, there are no standard errors in these periods.

The total PGG contribution, total support for the system, group profit, and system’s surplus were calculated for each period (see Fig. 3). The analyses for the second half were conducted. Support for the system was higher in the 1To2 and 2To1 conditions than in the 1ONLY condition (1To2vs1ONLY, *U* = 0, *p* < 0.001; 2To1vs1ONLY, *U* = 0, p < 0.001). There was no significant difference between the 2To1 and 1To2 conditions (*U* = 36, *p* = 0.957). We observed the same tendency in the PGG contribution: it was higher in the 1To2 and 2To1 conditions than in the 1ONLY condition (1To2vs1ONLY, *U* = 0, *p* < 0.001; 2To1vs1ONLY, *U* = 5, *p* < 0.001), but there was no significant difference between the 2To1 and 1To2 conditions (*U* = 36, *p* = 1.00). Unlike Experiment 1, the priority of the first- and second-order punishment did not matter to induce support and high PGG cooperation in Experiment 2. These results clearly reveal that to achieve high PGG cooperation, second-order punishment matters, but the importance of the priority of the first- and second-order punishment depends on the PR.

**Figure 4:**
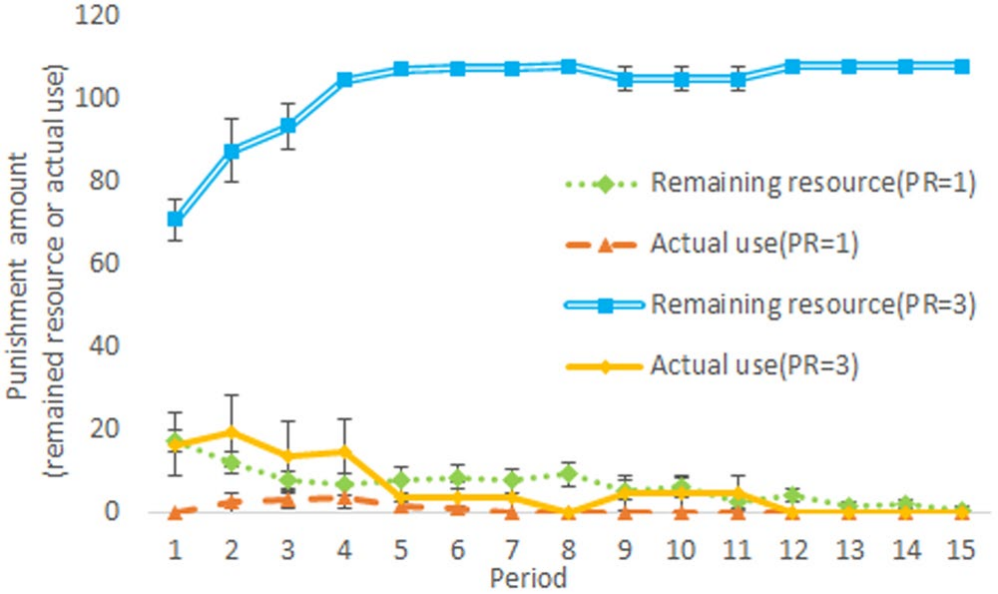
Remainder of punishment resources before second-order punishment and actual use of second-order punishment under the 1To2 condition in Experiments 1 (PR = 1) and 2 (PR = 3) (error bars denote standard errors).

Next we performed an analysis to understand why the 1To2 condition in Experiments 1 (PR = 1) and 2 (PR = 3) was completely different regarding members’ choices. Figure 4 shows the punishment resources before the second-order punishment, that is, the remaining resources after the first-order punishment, and actual use for the second-order punishment in the 1To2 conditions. The punishment resources were richer in Experiment 2 than in Experiment 1 in the first half (*U* = 0, *p* < 0.001) and second half (*U* = 0, *p* < 0.001) and the punishment use was higher in Experiment 2 in the first half (*U* = 11, *p* = 0.011). These results suggest that because of richer punishment resources, nonsupporters were punished more frequently in Experiment 2 than in Experiment 1. This is because the punishment resources in Experiment 2 were three times as high as in Experiment 1 even with the same support amount. Thus, the systems in Experiment 2 could keep more residue of punishment resources than those in Experiment 1 could after the systems punished noncooperators (see the Supplementary analysis for further explanation).

Lastly, the group profits were not different among conditions (1To2vs1ONLY, *U* = 33, *p* = 0.980; 1To2vs2To1, *U* = 35, *p* = 0.957; 1ONLYvs2To1, *U* = 35, *p* = 0.345). The system surplus was higher in the 1To2 and 2To1 conditions than in the 1ONLY condition (1To2vs1ONLY, *U* = 0, *p* < 0.001; 2To1vs1ONLY, *U* = 0, *p* < 0.001), but there was no significant difference between the 2To1 and 1To2 conditions (*U* = 35, *p* = 1.00), as both 1To2 and 2To1 conditions could induce stable support by members.

## Discussion

In these experiments, we focused on the priority of first- and second-order punishment in pool punishment systems. The results of Experiment 1 revealed that placing priority on the second-order punishment is critical for establishing cooperation in the PGG. As discussed in the introduction, the pool punishment system with only second-order punishment is not a social dilemma game, but a coordination game, and thus, full support by all members forms one of the equilibria. The results of the 2ONLY condition in Experiment 1 suggest that the risk-dominant equilibrium, that is, full support for the system by all members, was more likely to be chosen than the other equilibria. In the 2To1 condition, the pool system could punish noncooperators by using the residue of punishment resources, because the number of nonsupporters was relatively small. By contrast, in the 1To2 condition, the second-order free-rider problem occurred: the system could not punish noncooperators, because of a shortage of support by the members. The original purpose of the pool punishment system was to establish high cooperation in the PGG, and to achieve this purpose, the punishment not of noncooperators in the PGG but of nonsupporters of the punishment system should be prioritized. This conclusion seems to be counter-intuitive.

In Experiment 2, in which the punishment resources are abundant (PR = 3), the effect of the priority disappeared. High cooperation was achieved regardless of the priority of the first- and second-order punishment. This was because under abundant punishment resources, the nonsupporters could be punished even after the first-order punishment, and thus, the priority was no longer critical. The results of Experiments 1 and 2 suggest that the priority is nonnegligible when the punishment resources are scarce. We can claim that the priority of second-order punishment in the pool system is applicable to a wider range of real life, in which resources are usually scarce. For example, when the members of a group do not have sufficient resources to support the system, the system tends to lack the resources to punish noncooperators even if the PR is not small. Alternatively, when the default of cooperation level is quite low in a group, the pool system needs a large amount of resources to punish noncooperators and tends to lack such resources. Even in those cases, the pool system that gives priority to second-order punishment will be more likely to obtain high and general support and to achieve high cooperation in the PGG by effective punishment. When we intend to implement the pool punishment system to achieve high cooperation in real life, it is worth examining the priority of first- and second-order punishment.

To explore the further possibilities of this research, we clarify the points that concern the surplus of the system itself. As some individual, presumably a governor, such as a king or queen, has governed actual pool punishment systems historically, she/he might be interested in the surplus of the system, which could be his/her economic base. Our results suggest that the system that prioritizes second-order punishment obtains higher surplus than the system that prioritizes first-order punishment. Hence, the rational selfish king or queen would choose 2To1 over 1To2, and therefore, a more socially efficient system could be chosen by him/her. It is interesting to mention the possibility that pursuing private interest could promote the public interest. Of course, a selfish governor also has an incentive to choose 2ONLY. In Experiment 1, the system with 2ONLY receives as much profit as does the system with 2To1. In the system with 2ONLY, group members do not cooperate but support the system to be unpunished so that only the governor benefits. In other words, the pool punishment system has the potential risk of leading to tyranny. Recently, Ozono *et al*.^25^ conducted an experiment in which the pool punishment system was governed by a participant and he/she could freely punish group members. The authors found that some governors of the system punished both first- and second-order free riders spontaneously and the group resulted in high cooperation, but other governors punished only nonsupporters and received a certain benefit. This finding is consistent with the arguments presented earlier in this section.

Now, we consider an institution selection problem from the viewpoint of both the governors and governed. Hilbe *et al*.^26^ examined whether participants chose a pool punishment system with or without second-order punishment, using a “voting with feet” paradigm, in which each participant could freely choose and change the system to which they belonged. The authors revealed that the participants tended to choose the punishment system without second-order punishment, especially at the beginning of the experiment. If both the governed and governor of the system could choose punishment system rule, the system with second-order punishment (in our experiment, the 2To1 condition) might be chosen more than that without second-order punishment (in our experiment, the 1ONLY condition), because the former is more beneficial for governors. The governors, however, have an incentive to choose 2ONLY, so if the governed cannot migrate easily, tyrannical states are more likely to emerge. As far as we know, no prior study has investigated the dynamic process of system choice by both governors and the governed, and this is a promising approach to study the development of punishment systems in real life.

Another extension of the present study would be to investigate pool punishment regimes by integrating recent developments of social dilemma studies. Some studies focus on the solution of first-order or second-order free-rider problems from different perspectives^27–36^. In particular, it has been shown recently that taking spatial structure into account is important to understand the evolution of punishment^29–36^. As a pool punishment system works under a structured space, pool punishment systems that are effective for public goods provision are contingent on spatial structures. Hence, studying the relationship between spatial structures and effective pool punishment systems could be an interesting future research direction.

## Methods

The Waseda University Ethical Review Board approved this study. The methods were carried out in accordance with the approved guidelines. Written informed consent was obtained from all subjects prior to beginning the experiment.

### Participants

We recruited 172 undergraduate students in Experiment 1 and 116 undergraduate students in Experiment 2 from various disciplines using a university portal website. In each session, there were 8–16 participants. Each session was assigned to one of the four conditions (Experiment 1) and three conditions (Experiment 2). Participants were randomly separated into groups of four, and group members were fixed throughout the duration of the experiment according to a partner-matching design. In Experiment 1, 44 participated in 1To2 (11 groups), 52 in 2To1 (13 groups), 40 in 1ONLY (10 groups), and 36 in 2ONLY (9 groups). In Experiment 2, 36 participated in 1To2 (9 groups), 40 in 2To1 (10 groups), and 40 in 1ONLY (10 groups).

### Procedure

In all conditions, participants were assigned to laboratory booths to ensure their anonymous and independent decisions. After reading explanations, participants were questioned on the experiment details. All participants completely understood the rules of all transactions and could calculate their payoffs. Participants were then randomly and anonymously allocated to groups of four, and these groups played the PGG and punishment stages (a detailed account of these stages is given below). The participants knew that the group composition would be unchanged throughout the experiment, that the periods would be repeated 15 times, and that tokens earned during transactions would be redeemed for money.

#### PGG stage

Each of the four members was given 20 tokens at the beginning of the stage, and simultaneously chose his/her contribution from 0 to 20 in increments of 1, which was subtracted from his/her endowment of 20 tokens. The total tokens each member contributed were multiplied by 1.6 and distributed equally to the group members.

#### Pool punishment system stage

Each member was given another 9 tokens at the beginning of the stage. Each member simultaneously decided how many tokens he/she would provide (support) to his/her pool punishment system from 0 to 9. In Experiment 1, total tokens provided to the system became the punishment resources of the system and in Experiment 2, total provided tokens were multiplied by three, becoming the punishment resources of the system.

The punishment rule varied among conditions. In the 1ONLY condition, the first-order free riders—less cooperators—were punished in the following order. First, the least cooperator’s tokens were reduced to zero. The second least, third least, and fourth least cooperators were punished in that order. If there were more than two members who contributed the same amount, the order was determined at random. A full cooperator, who contributed 20 tokens, was never punished. When the system reduced one member’s tokens to zero, the punishment resources of the system also decreased by the same amount. When the system cannot reduce the tokens of the member to 0 because of the shortage of punishment resources, the system reduced as many tokens as possible. After the punishment resources become 0, the system cannot punish any more.

In the 2ONLY condition, second-order free riders—less supporters—were punished in the following order. First, the least supporter was punished. Next, the second least, third least, and fourth least supporters were punished in that order. Except for the order of being punished, the punishment rule was the same as in the 1ONLY condition.

In the 1To2 condition, the first-order punishment was executed first followed by the second-order punishment. In the 2To1 condition, the second-order punishment was executed first, followed by the first-order punishment. The punishment rule was the same in these conditions.

The PGG results were provided to all members after the decision of support. We applied Traulsen *et al*.’s^17^ assumption that the nature of the pool punishment system is to decide whether to support the punishment organization to establish the organization before the results of the PGG. All members were informed who had been punished and by how much immediately after the decision of support for the system (see the supplementary method).

These two stages were repeated 15 times. We used z-Tree software^37^ to conduct the experiments. Each session took approximately 70 minutes to complete on average. The total attained score was converted to money using the rate 1 token = 2.5 yen (100 yen = +−1 US dollar), and a 500-yen show-up fee was given to participants who concluded the experiment. The average remuneration was 1,580 yen.

### Data availability

All data generated or analyzed during this study are included in the Supplementary Information files.

## Electronic supplementary material

Supplementary Information

Dataset 1
