# Supplementary material for: Punishing second-order free riders before first-order free riders: The effect of pool punishment priority on cooperation
Source: Sci Rep. 2017 Oct 30;7:14379. doi: 10.1038/s41598-017-13918-8 (PMC5662696; doi:10.1038/s41598-017-13918-8)
Supplement: Supplementary file 1 — Supplementary Information [file 41598_2017_13918_MOESM1_ESM.pdf]

Supplementary Information

Punishing second-order free riders before first-order free riders: The effect of pool punishment priority on cooperation

Hiroki Ozono, Yoshio Kamijo, and Kazumi Shimizu

Supplementary analysis

<u>The reasons for the abundance of the amount of punishment resources for the second-order punishment in Experiment 2.</u>

There are several reasons for the abundance of the amount of punishment resources for the second-order punishment in Experiment 2. First, as indicated in the main text, the punishment resources in Experiment 2 are three times as much as in Experiment 1, even if the support amount is the same, and thus, the systems in Experiment 2 can keep more residue of punishment resources than can the systems in Experiment 1 after the systems have punished noncooperators. The second reason is encapsulated by the following concept: "As the punishment resources are rich, I will be punished if I don't cooperate." Since the PR=3 condition correlates the current choice with the future (possible) punishment, it can enhance cooperation in the current period. As a result, the cost for first-order punishment might be less in Experiment 2 than in Experiment 1 and more resources can be used for second-order punishment in Experiment 2. The data support this argument. The PGG contribution was higher in Experiment 2 than in Experiment 1, both in the first half ($p<.001$) and second half ($p<.001$). In addition, the PGG contribution in the first period in Experiment 2 was higher than in Experiment 1 ($p=.016$), which suggests that the participants in Experiment 2 expected the first-order punishment to be more probable before the game started. Third, the punishment resources for the second-order punishment remained rich because of the high level of cooperation in the PGG, and thus, the participants supported the system more to avoid the second-order punishment and the punishment resources increased. Actually, the support amount as an absolute value was higher in Experiment 2 than in Experiment 1 both in the first half ($p<.001$) and second half ($p<.001$). While the support amount in the first period was not different between Experiments 1 and 2 ($p=.12$), the participants in Experiment 2 gave more support in the second period than in the first period (Wilcoxon matched-pairs signed-rank test: $p=.047$). By contrast, the participants in Experiment 1 seemed to give less support in the second period than in the first period (Wilcoxon matched-pairs signed-rank test: $p=.063$). These results suggest that the participants in Experiment 2 might realize the second-order punishment would be executed after experiencing the first period but the participants in Experiment 1 might not think so. For the abovementioned three reasons, as for the 1To2 conditions, the second-order punishment was more effective and the members were more cooperative in Experiment 2 than in Experiment 1.

Supplementary method

1. <u>Instruction of the experiment</u>

After a brief verbal introduction, participants read the following instructions on the computer monitor telling them that they would take part in an experiment on decision making.

General Guidance

This is an experiment about decision making. You will be paid for participating, and the amount of money you will earn depends on the decisions that you and the other participants make. At the end of today's session, you will be paid privately and in cash for your decisions.

You will never be asked to reveal your identity to anyone during the course of the experiment. Your name will never be associated with any of your decisions.

At this time, you will be given 500 yens (= 5–6 dollars) for coming on time. All the money that you earn after this experiment will be yours to keep.

Earnings

In this experiment, you are in a group of size 4 (you plus 3 others) and you will be asked to make a series of choices about how to allocate a set of tokens. You and the other participants have been randomly assigned to the group, and you *will not* be able to know each other's identities. However, the group members will remain the same throughout the experiment.

The details of the experimental transactions are as follows. Four members named A, B, C, and D will play the same role. The experiment comprises two stages, the 1st and 2nd stages. These stages will be repeated 15 times, and the tokens you earn during transactions will be redeemed as monetary remuneration.

Now, let us explain the details of each stage.

1st stage

Each of the four members contributes 20 tokens at the beginning of this stage. The members are asked to decide how many tokens to contribute to the group pool. You lose the amount you contribute to the pool, but 40% of the sum of the tokens is given to each of the 4 members, including you. Hence, the number of tokens you contribute and the sum of tokens contributed by any participant, including you, will determine the payoff you receive. Each choice that you make is similar to the following example.

-Examples of choices you will make in the 1st stage and earnings

Example 1: Suppose that you and the other 3 members all contribute 20 tokens to a pool. You will earn:

20 (initial endowment) − 20 (the tokens you contributed)

+ 0.4 * 80 (the sum of tokens the 4 members contributed)

=32

Example 2: Suppose that you and the other 3 members all contribute nothing. You will earn:

100 (initial endowment) − 0 (the tokens you contributed)

+ 0.4 * 0 (the sum of tokens the 4 members contributed)
=20
Example 3: Suppose that you give 4 tokens and the other members contribute 5, 10, and 16 tokens each. You will earn:
20 (initial endowment) − 4 (the tokens you contributed)
+ 0.4 * 35 (the sum of tokens the 4 members contributed)
=30

2nd stage (1TO2 condition with PR=1):

Each of the four members are given another 9 tokens at the beginning of this stage. The members are asked to decide how many tokens to provide to the “reduction system” from 0 to 9. The total tokens provided to the system became the “reduction resources” of the system.The system reduces the members in the order of the following two steps.

Step 1

In Step 1, first, the least contributor for the group pool in the 1st stage just before this 2nd stage reduces his/her tokens to 0. Next, the second least, third least, and fourth least contributors are reduced in that order. If there are more than two members who contribute the same amount, the order is determined at random. A full contributor, who contributes 20 tokens, is never punished. When the system reduces one member’s tokens to 0, the reduction resources of the system also decrease by the same amount. For example, imagine a situation in which the initial reduction resources of the system is 40 and the least contributor has 28 tokens before being reduced. When the least contributor is punished, his/her tokens are reduced from 28 to 0 by the system and the reduction resources decrease from 40 to 12 (=40−28) tokens. When the system cannot reduce the tokens of the member to 0 because of the shortage of reduction resources, the system reduces as many tokens as possible. For example, imagine a situation in which the initial punishment resources of the system is 10 and the least contributor has 22 tokens before being reduced. When the least contributor is reduced, his/her tokens are reduced from 22 to 12 (=22−10) by the system and the reduction resources decrease from 10 to 0 (=10−10) tokens. After the reduction resources become 0, the system can no longer reduce the members’ tokens.

Step 2

In Step 2, less providers for the reduction system in this 2nd stage reduce their tokens if reduction resources are not drained in Step 1. The system reduces the members’ tokens in the following order. First, the least provider is punished. Next, the second least, third least, and fourth least providers are punished in that order. Except for the order of being reduced, the reduction rule is completely the same as in Step 1; that is, less providers reduce their tokens to 0 and full providers who provided 9 tokens to the system are never reduced.

Examples of choices you will make in 2nd stage and earnings

Suppose that the contributions of A, B, C, and D in the 1st stage are 10, 2, 8, and 0, respectively, and that the provision to the reduction system in the 2nd stage is 9, 7, 9, and 8, respectively. In this case,

the total amounts each member receives before the reduction in step 1 are 18, 28, 20, and 29, respectively, and the total provisions to the reduction system (= reduction resources) is 33 (=9+7+9+8).

In Step 1, first, D is the least contributor, and thus, D's tokens are reduced from 29 to 0 (=29 − 29). The system use 29 tokens to reduce D's tokens and the reduction resources become 4 (=33 − 29). Second, B is the second least contributor. The system does not have enough reduction resources to reduce all of B's 28 tokens, and thus, the system reduces as many tokens as possible. As a result, B's tokens reduce from 28 to 24 (=28 − 4).
The reduction resources become 0 (=4 − 4), and thus, the system can no longer reduce the other members.

In this case, the system cannot reduce in Step 2, because there are no reduction resources. However, if the reduction resources are not drained in Step 1, then next, less providers for the reduction system in this 2nd stage reduce their tokens.

Feedback
The results of stage 1, that is, how many tokens each member contributed to the group pool, are provided to all members after the decision of provision for the system in stage 2. All members are informed about who has reduced tokens and by how much immediately after the decision of provision for the reduction system.

These two stages are repeated 15 times. The total attained score is converted to money using the rate 1 token＝0.7 yen, and the converted amount plus 500 yen (the show-up fee) is given to you at the end of this experiment.

After this general instruction is given, all participants start the experiment after filling out a confirmation test.

Confirmation Test
Before you start to make your decision, you should solve all questions in the paper. Read carefully through the provided information provided and write down the number of points in the paper. We will watch you solving the examples, check whether you get the right answers, and help you if you have a problem or question.

Before the decision making
Good, now everybody has solved the problems. If anybody has any more questions, raise your hand now. Otherwise let's practice how to input your decision on your computer screen.

2. Screen shots of computer displays during the experiment.

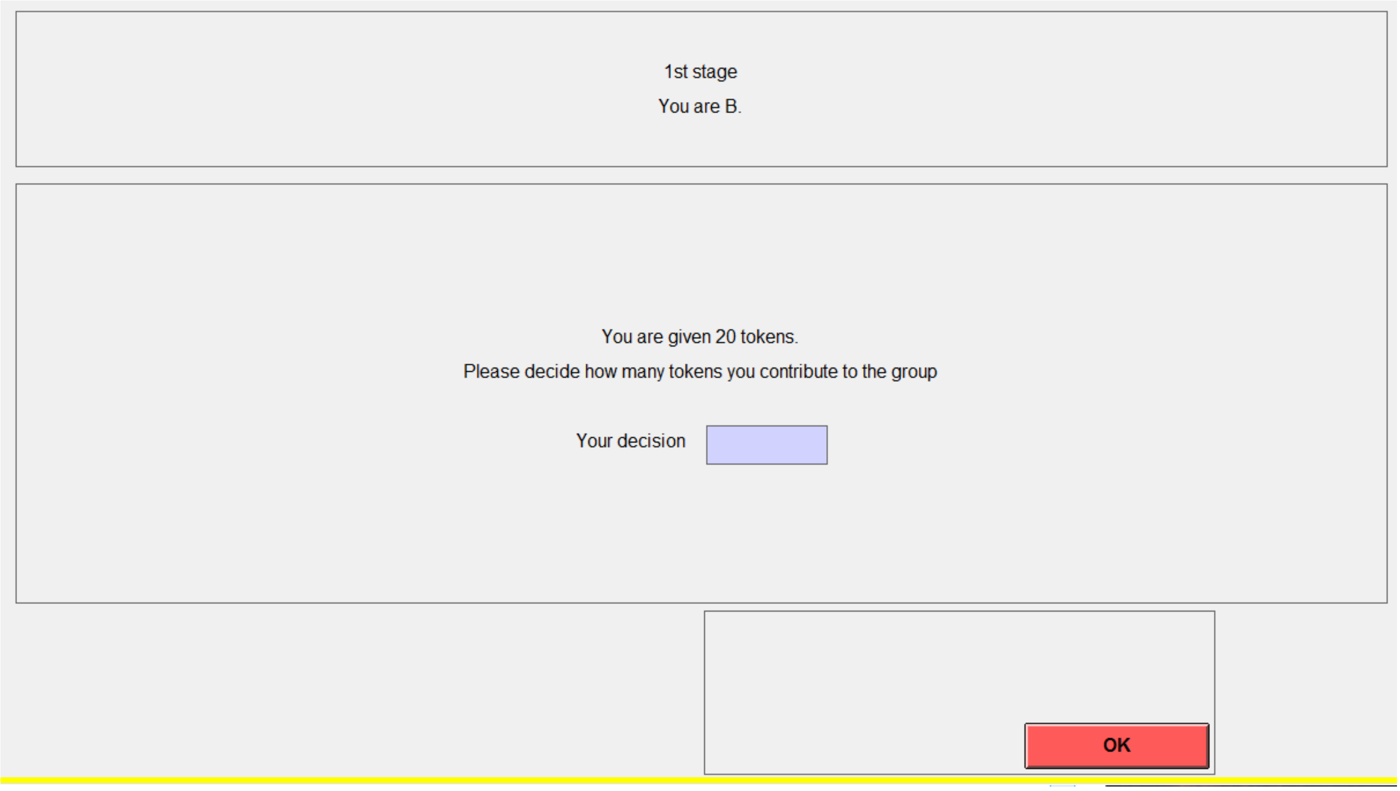

Screen shot of computer display when the participants make decisions in the 1st stage.

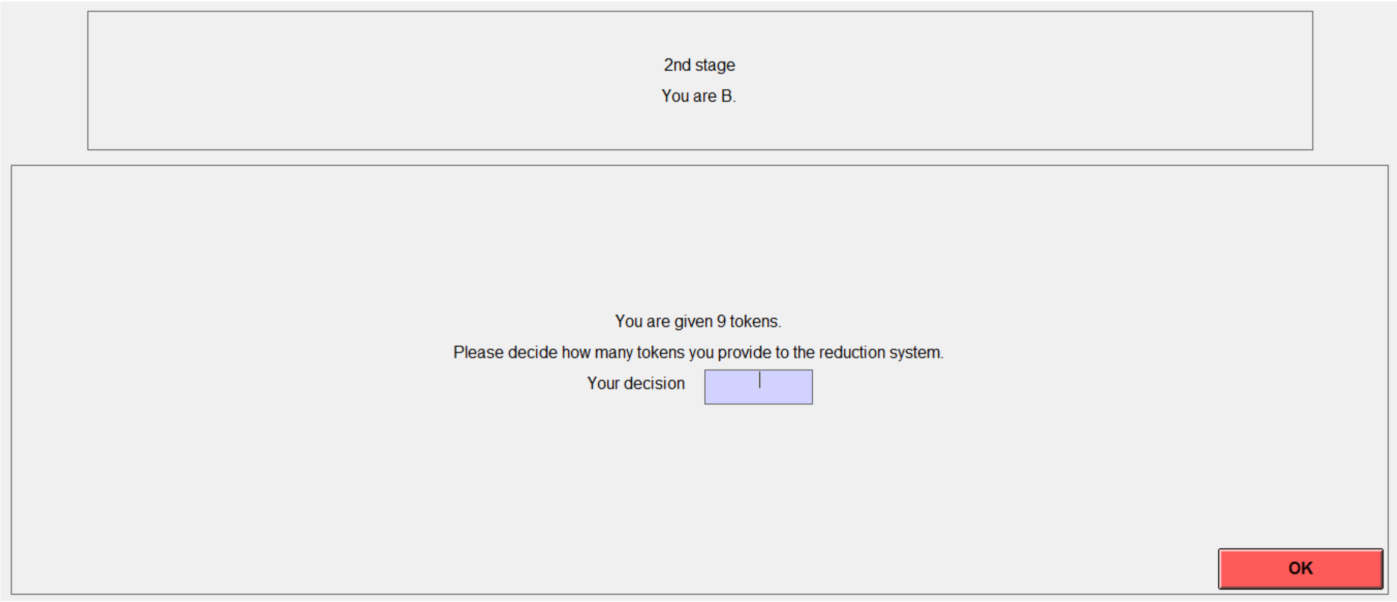

Screen shot of computer display when participants make decisions in the 2nd stage.

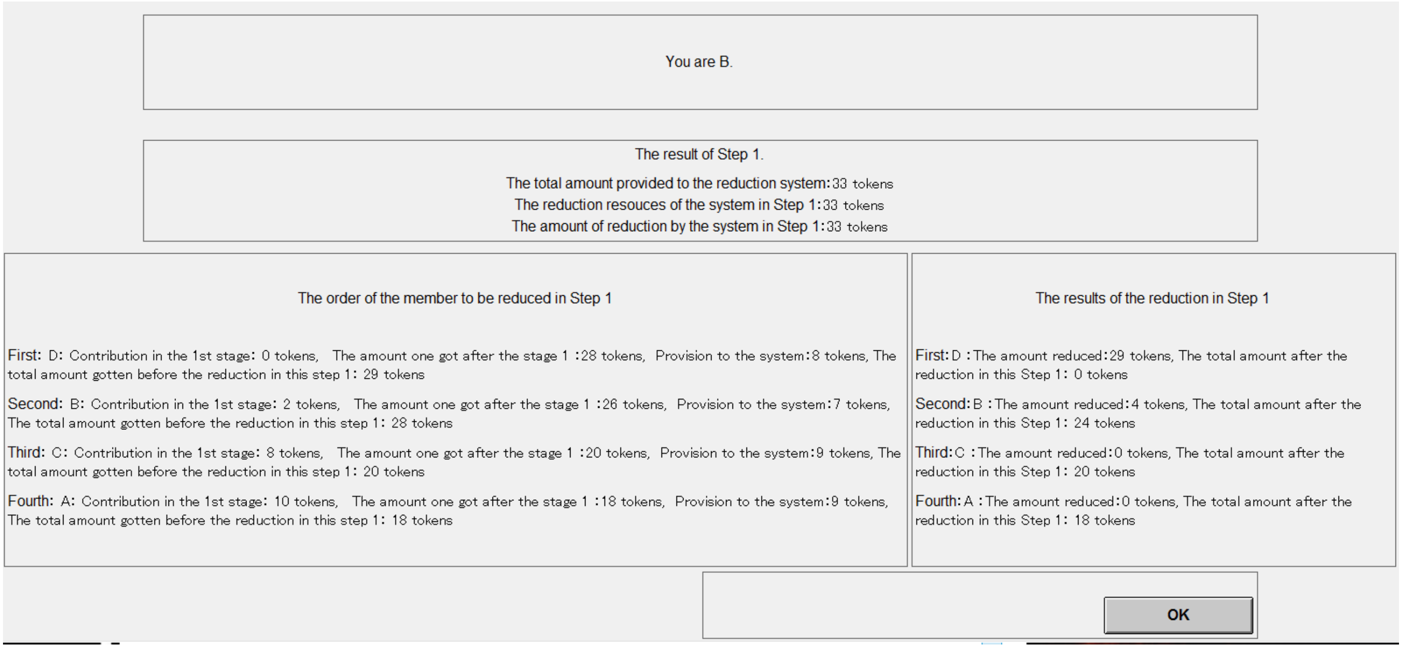

Screen shot of computer display when showing feedback of Step 1 of the 2nd stage.

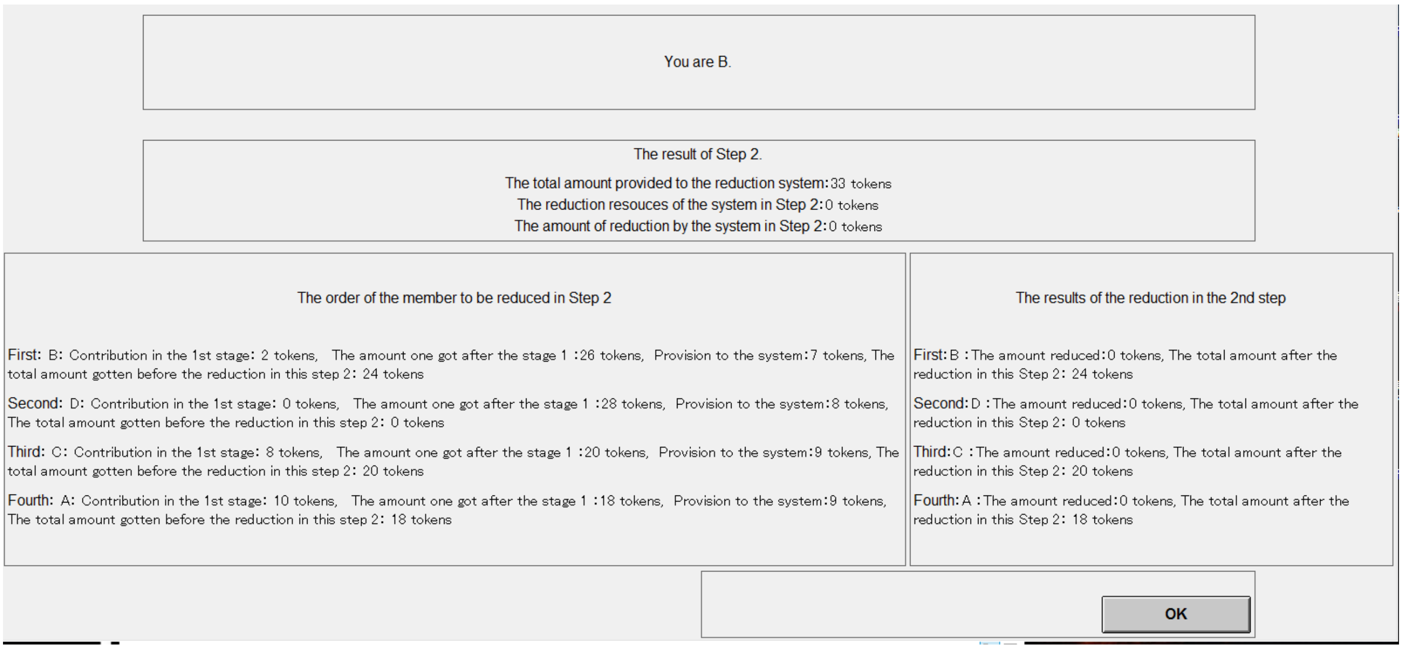

Screen shot of computer display when showing feedback of Step 2 of the 2nd stage.
